# Production of ERCP training model using a 3D printing technique (with video)

**DOI:** 10.1186/s12876-020-01295-y

**Published:** 2020-05-11

**Authors:** Chang-Il Kwon, Yeonsun Shin, Jaeok Hong, Minje Im, Guk Bae Kim, Dong Hee Koh, Tae Jun Song, Won Suk Park, Jong Jin Hyun, Seok Jeong

**Affiliations:** 1 Digestive Disease Center, CHA Bundang Medical Center, CHA University School of Medicine, Seongnam, South Korea; 2 Research Group for Endoscopic Instruments and Stents, Korean Society of Gastrointestinal Endoscopy, Seoul, Korea; 3 Gluck, Seoul, South Korea; 4 Anymedi Inc., Seoul, South Korea; 5 Division of Gastroenterology, Department of Internal Medicine, Hallym University Dongtan Sacred Heart Hospital, Hallym University College of Medicine, Hwaseong, South Korea; 6 Division of Gastroenterology, Department of Internal Medicine, Asan medical center, Ulsan University College of medicine, Seoul, South Korea; 7 Division of Gastroenterology, Department of Internal Medicine, Daejeon St. Mary’s Hospital, College of Medicine, The Catholic University of Korea, Daejeon, South Korea; 8 Division of Gastroenterology and Hepatology, Korea University College of Medicine, Seoul, South Korea; 9 Division of Gastroenterology, Department of Internal Medicine, Inha University Hospital, Inha University School of Medicine, 27 Inhang-ro, Jung-gu, Incheon, 22332 Republic of Korea

**Keywords:** Printing, Three-dimensional, Cholangiopancreatography, endoscopic retrograde, Endoscopy, Training model

## Abstract

**Background:**

ERCP training models are very different in terms of anatomical differences, ethical issues, storage problems, realistic tactile sensation, durability and portability. There is no easy way to select an optimized model for ERCP training. If the ERCP training model could be made as a soft silicone model using 3D printing technique, it would have numerous advantages over the models presented so far. The purpose of this study was to develop an optimized ERCP training model using a 3D printing technique and to try to find ways for implementing various practical techniques.

**Methods:**

All organ parts of this model were fabricated using silicone molding techniques with 3D printing. Especially, various anatomy of the ampulla of Vater and common bile duct (CBD) were creatively designed for different diagnostic and therapeutic procedures. In order to manufacture each of the designed organ parts with silicone, a negative part had to be newly designed to produce the molder. The negative molders were 3D printed and then injection molding was applied to obtain organ parts in silicone material. The eight different types of ampulla and CBD were repeatedly utilized and replaced to the main system as a module-type.

**Results:**

ERCP training silicone model using 3D technique was semi-permanently used to repeat various ERCP procedures. All ERCP procedures using this model could be observed by real-time fluoroscopic examination as well as endoscopic examination simultaneously. Using different ampulla and CBD modules, basic biliary cannulation, difficult cannulation, stone extraction, mechanical lithotripsy, metal stent insertion, plastic stent insertion, and balloon dilation were successfully and repeatedly achieved. Endoscopic sphincterotomy was also performed on a specialized ampulla using a Vienna sausage. After repeat procedures and trainings, all parts of organs including the ampulla and CBD modules were not markedly damaged or deformed.

**Conclusions:**

We made a specialized ERCP training silicon model with 3D printing technique. This model is durable, relatively cheap and easy to make, and thus allows the users to perform various specialized ERCP techniques, which increases its chances of being a good ERCP training model.

## Background

Endoscopic retrograde cholangiopancreatography (ERCP) is an attractive procedure for endoscopists because it provides a variety of treatment options and clinically dramatic results [1]. However, because the techniques of ERCP are so diverse and complex, the process of learning them is very difficult and time-consuming [2]. In addition, it is necessary to proceed very sophisticatedly and carefully because it can induce serious adverse events even during the basic procedures. Therefore, both patients and endoscopists are usually under great pressure when ERCP is being conducted. To minimize this burden, endoscopists should perform all techniques of ERCP in a highly skilled and well-educated state to prevent all procedural-related adverse events. Although there is no qualifying test for ERCP procedures, some criteria or proposed curriculum for evaluating the ability to perform ERCP have been suggested and referenced in the ERCP training [3–5]. However, under the mentorship program, both trainees and mentors are forced to undertake ERCP education with great burdens and limitations.

For these reasons, ERCP training models have been developed and applied in various ways for a long time. ERCP training models have been developed using live animal models, ex vivo porcine models, computer simulators, and mechanical methods [6]. Because each method is very different in terms of anatomical differences, ethical issues, storage problems, realistic tactile sensation, durability and portability, there is no easy way to select an optimized model for ERCP training [7].

With the development of endoscopic accessories using three-dimensional (3D) printing, endoscopic training models have also been introduced [8–12]. However, products made by 3D printing technique are relatively hard and not suitable for the endoscopic training model. In recent years, the 3D printing technique has been used to create a platform or mold, in which silicone is then poured or inserted to create the desired soft model, such as endoscopic biopsy model and percutaneous endoscopic gastrostomy model [13, 14]. If the ERCP training model could be made as a soft silicone model, it would have numerous advantages over the models presented so far, including the possibility of practicing various techniques with it, and its ability to withstand repeated use.

The purpose of this study was to develop an optimized ERCP training silicone model using 3D printing technique and to try and determine ways to implement various practical techniques.

## Methods

### 3D modeling and fabrication of ERCP training phantom

In order to produce an ERCP phantom model, same data of computed tomography gastrography (CTG), used in previously reported studies [15, 16], were used retrospectively to accurately understand and mimic the interior space and path of the stomach and duodenum. This study was approved by CHA Bundang Medical Center Institutional Review Board (No. CHAMC 2019–08-025).

**Fig. 1:**
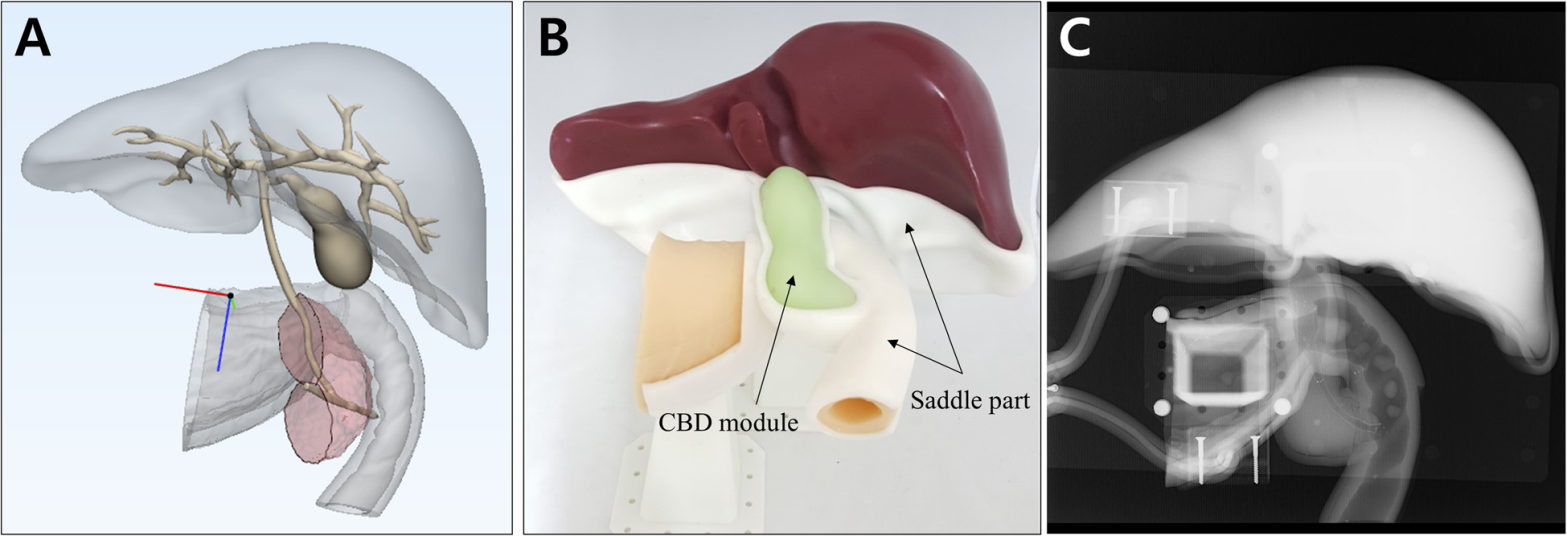
3D modeling and fabrication of ERCP training simulator. **a** 3D model parts; **b** Fabricated 3D phantoms. ‘CBD module’ refers to a removable ampullary and common bile duct part. The ERCP phantom is fixed with a supporting stand and positioned to the prone position; **c** Fluoroscopic image of the phantoms

**Fig. 2:**
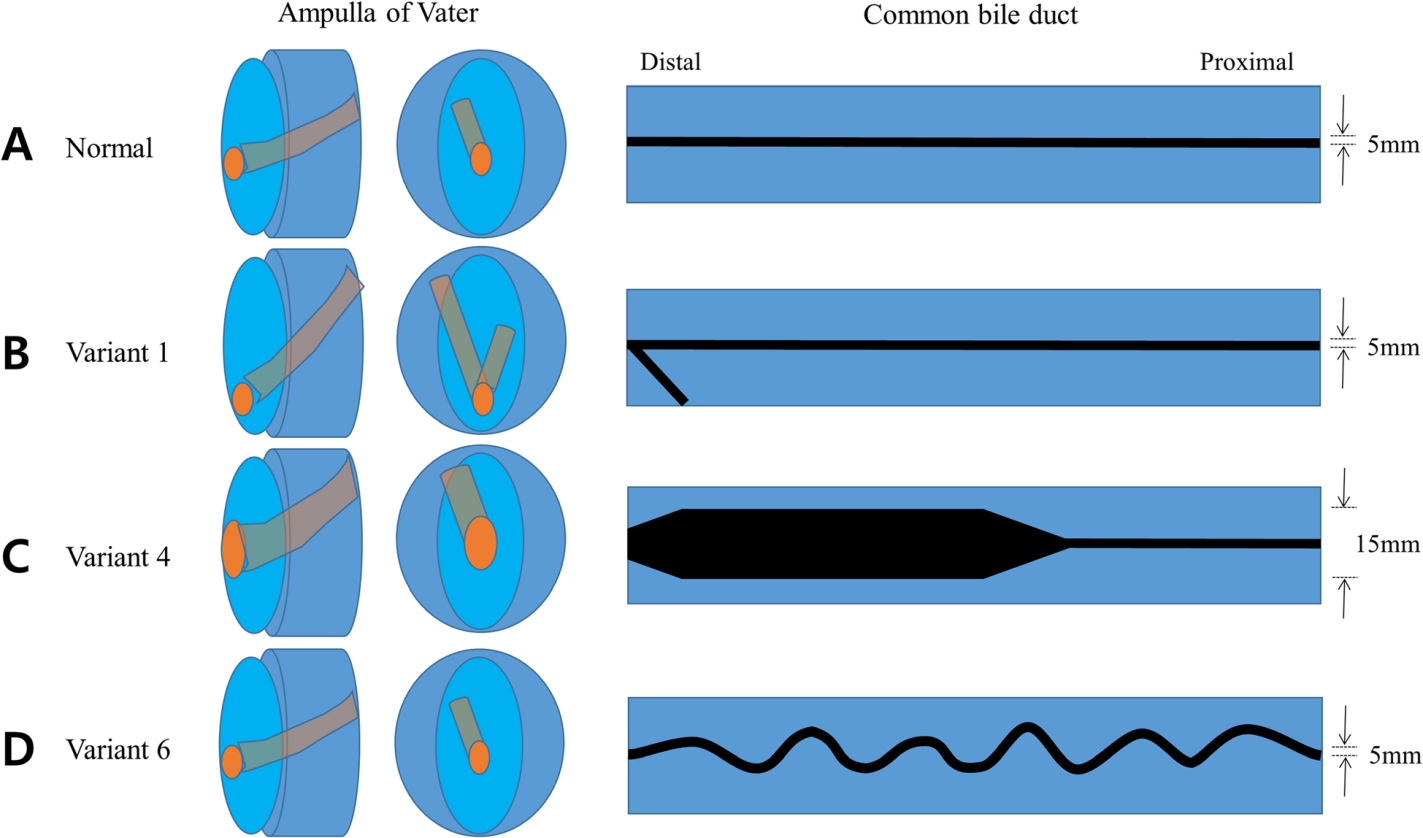
Concepts for the ampulla of Vater and common bile duct (CBD) shapes. **a** Normal type: The ampulla orifice is located in the middle of the area and the width is 5 mm. The CBD is located at 11 o’clock and the width remains constant at 5 mm; **b** Variant 1: The ampulla orifice is located at the very bottom and is designed to cause cannulation difficulty. A short pancreatic orifice is also created for double guidewire method; **c** Variant 4: The ampulla orifice is expanded to facilitate stone removal. The distal CBD is extensively expanded so that stone insertion and stone movement are free; **d** Variant 6. The width of the CBD is constant, but the direction is very wavy and is designed to induce difficult guidewire insertion

**Fig. 3:**
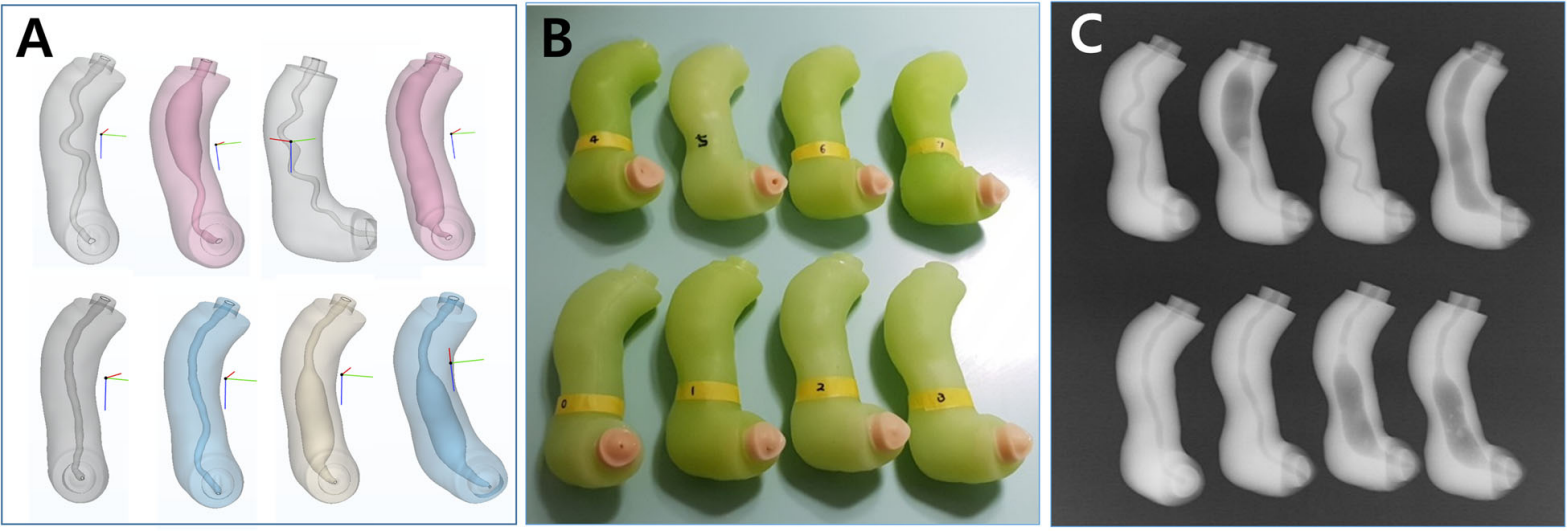
3D modeling and fabrication of eight different types of ampullary and CBD modules. **a** 3D model parts of 8 different types; **b** 8 fabricated CBD modules; **c** Fluoroscopic image of the modules

Digital 3D models of stomach and duodenum were obtained by importing the CT image data to the in-house software by StereoLithography (STL) file format. In addition, a hepatobiliary system, consisting of the ampulla of Vater, pancreatic duct with pancreas, common bile duct (CBD), gallbladder, liver, and intrahepatic bile ducts, was implemented in three dimensions using two open source programs of MeshLab and MeshMixer (Fig. 1a). In this study, endoscopic training in various anatomy of the ampulla of Vater and CBD had to be emphasized. Therefore, removable ampullary and CBD modules were mounted on the base of phantom (Fig. 1b and c). For performing different therapeutic procedures, eight different types of ampullary and CBD modules were separately designed to be utilized and repeatedly replaced on the base of phantom (Figs. 2 and 3).

The main parts produced in this phantom were the lower stomach and duodenum, the ampullary and CBD module, the liver having intra hepatic bile ducts, and finally the saddle that can stably support them. In this study, all organ parts were fabricated using silicone molding techniques with 3D printing, while only the saddle part was produced directly by the 3D printer (3DM Tough-3.6, 3DMaterials, Zeron-2500, Zeron, Korea). In order to manufacture each of the designed organ parts with silicone, a negative part had to be newly designed to produce the molder. The negative molders were 3D printed (3DM DW-06, 3DMaterials, Zeron-2500, Zeron, Korea) and then injection molding was applied to obtain organ parts in silicone material (Dragon Skin 10, Smooth-on, USA; shore A (hardness) = 10, elongation = 1000%).

## Results

### Position and orientation of the ERCP phantom

**Fig. 4:**
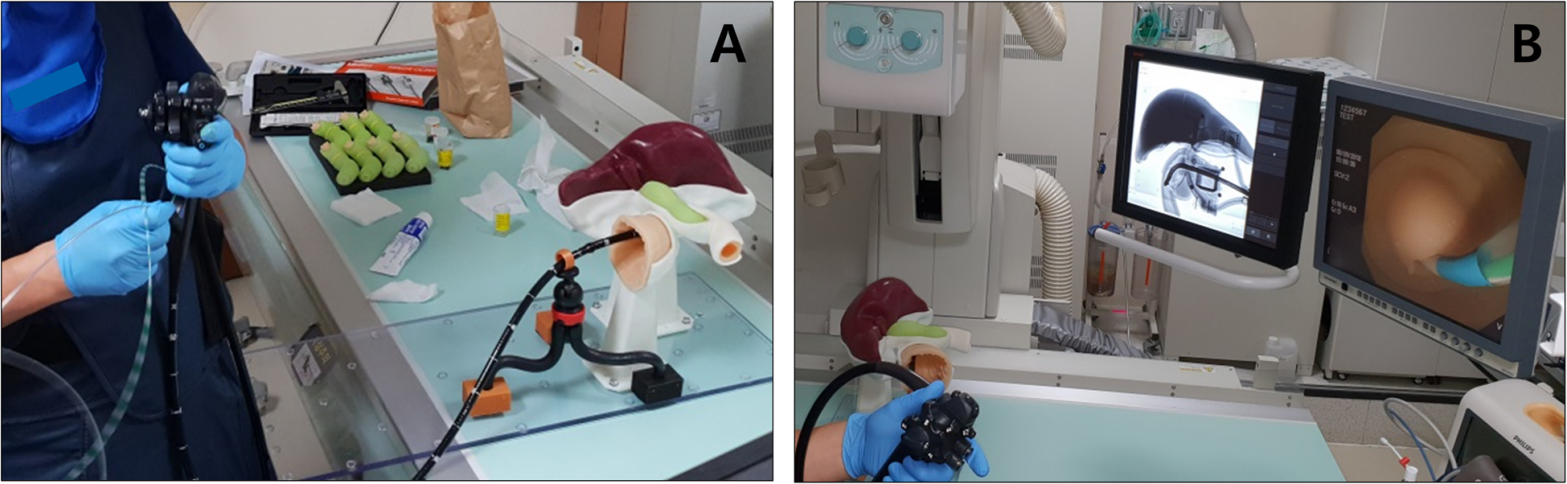
Orientation of the ERCP phantom. **a** The ERCP phantom is placed in the opposite direction on the fluoroscopy table; **b** Adjustment of the fluoroscopic imaging

For ERCP training, it is important to acquire real-time fluoroscopic images as well as endoscopic images. We fixed the ERCP phantom with a supporting stand, also made by the 3D printing technique, in the prone position as in a common ERCP procedure position (Fig. 1b). Because the shortening step was omitted during duodenoscope insertion into the area of ampulla, and the phantom was inversely located on the fluoroscopy table, it was necessary to adjust the direction of the fluoroscopic imaging (Fig. 4). It was not necessary to adjust the direction of endoscopic imaging.

### Implementation of ERCP procedures

**Fig. 5:**
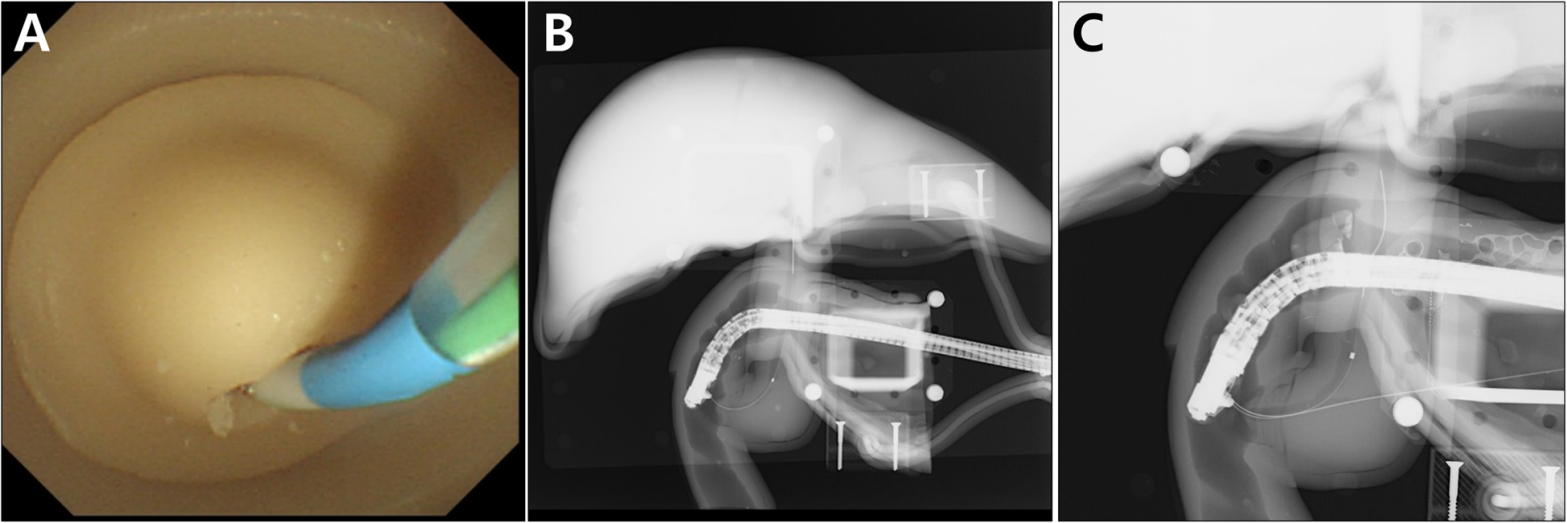
Biliary cannulation using the basic module. **a** Endoscopic view; **b** Fluoroscopic view; **c** Fluoroscopic view of double guidewire technique

Using the basic module, biliary cannulation was easily and repeatedly achieved with a guidewire (VisiGlide 2™, Olympus Co., Tokyo, Japan; or gSlider, Medwork GmbH, Aisch, Germany) and cannulation catheter (ERCP-catheter, MTW-Endoskopie, Wesel, Germany) (Fig. 5a and b). Biliary cannulation with double guidewire method and difficult cannulation were also successfully achieved using the CBD stricture modules (Fig. 5c). Despite repeated procedures, the ampulla portions of each module did not suffer damage or tears. Also, we did not need to repeat injecting the dye into the CBD modules due to excellent visualization of biliary tract under the fluoroscope.

**Fig. 6:**
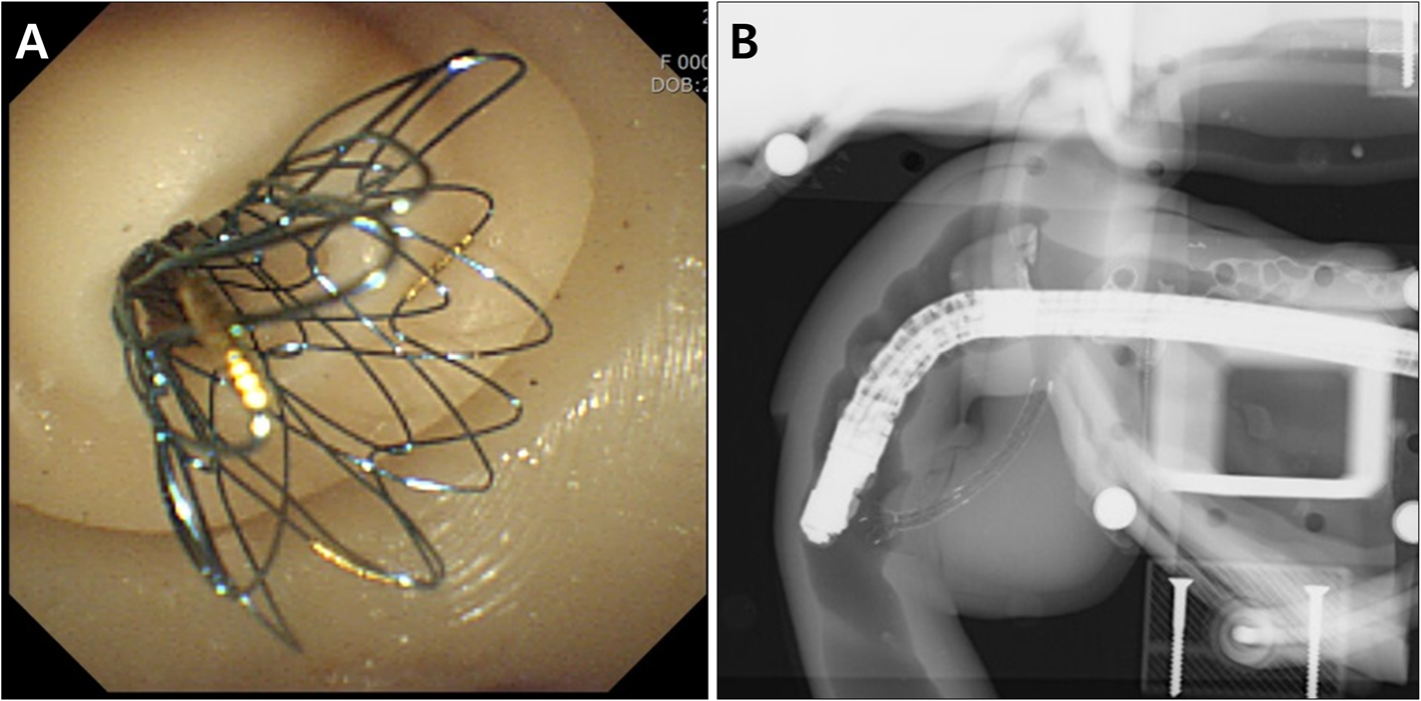
Biliary metal stent insertion using distal stricture and proximal dilation of the CBD module (**a** Endoscopic view; **b** Fluoroscopic view)

Biliary stent placement was implemented using the distal stricture and proximal dilation CBD module (Fig. 6). After the insertion of self-expandable metal stent (SEMS) (Hanarostent®, M.I.Tech, Pyeongtaek, Korea) through the stricture area, the SEMS was retrieved again, and was reinserted into the deploying catheter for repeated procedures. Plastic stent insertion and endoscopic balloon dilation were also successfully performed using the same module.

**Fig. 7:**
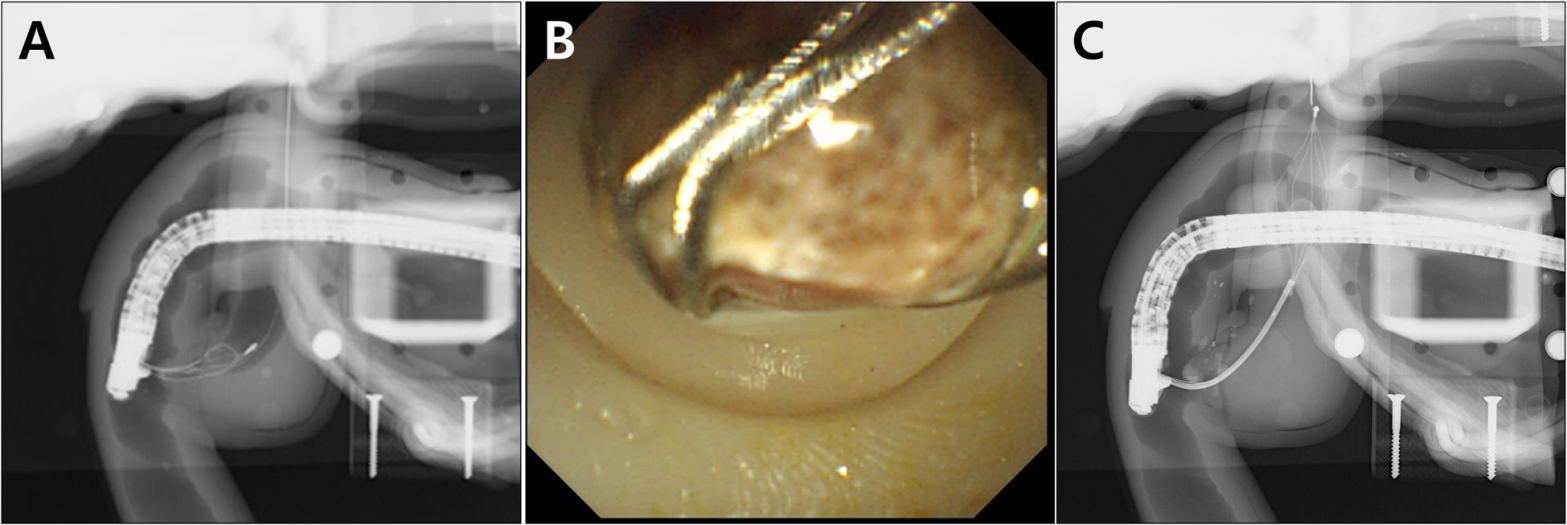
Biliary stone extraction and mechanical lithotripsy. **a** Fluoroscopic image of biliary stone extraction using the CBD dilation module; **b** Endoscopic image of biliary stone extraction without sphincterotomy; **c** Fluoroscopic image of mechanical lithotripsy using the distal stricture and proximal dilation of the CBD module

Bile duct stone extraction was also successfully implemented using the CBD dilation module. Before the procedure, we inserted a 12 mm cholesterol stone, into the module, which was obtained from a surgically resected human gallbladder. After capturing the stone by a conventional basket catheter (Flower Basket V™, Olympus Co.), the stone was pulled out from the ampulla by using a flip down technique (Fig. 7a and b). The ampullary orifice of the module was well-extended without sphincterotomy, and it was not damaged after the stone removal. Mechanical lithotripsy with a lithotripsy basket catheter (Trapezoid™ RX, Boston Scientific Co., Natick, MA.) was also successfully implemented using the distal stricture and proximal dilation CBD module (Fig. 7c).

**Fig. 8:**
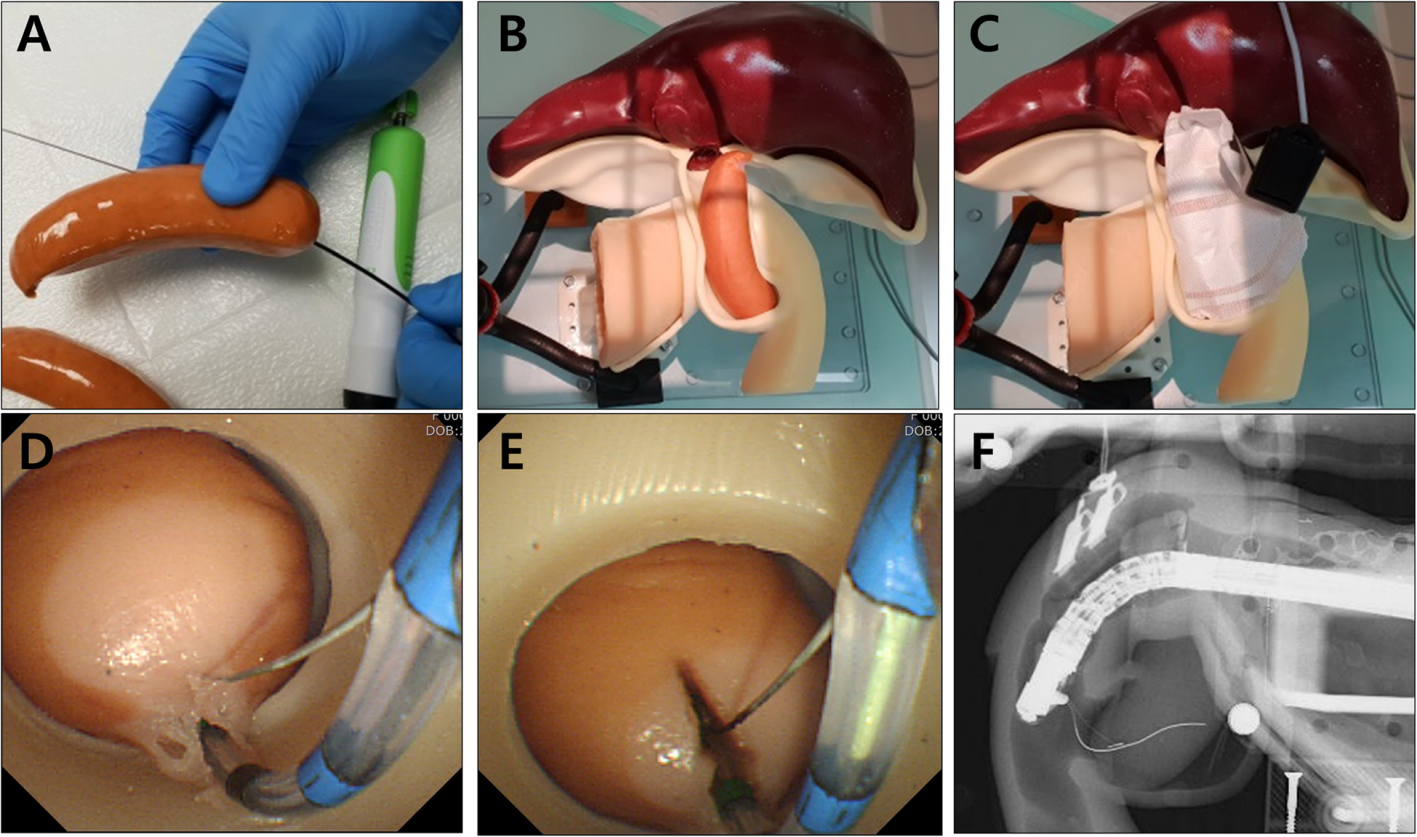
Endoscopic sphincterotomy using a Vienna sausage. **a** Making a small tract in a Vienna sausage as a vertical axis using an endoscopic ultrasound-guided biopsy needle; **b** Assembly of the sausage into the module area of the phantom; **c** Attachment of electrical plate to the sausage; **d** Endoscopic view of the exposed sausage at the ampullary area; **e** Endoscopic sphincterotomy after insertion of a guidewire and sphincterotome; **f** Fluoroscopic image of endoscopic sphincterotomy


Endoscopic sphincterotomy was performed on a specialized ampulla using a Vienna sausage (Fig. 8). Before the assembly of the sausage into the module area of the phantom, a small tract was made in the sausage using an endoscopic ultrasound-guided biopsy needle (Acquire™ needle, Boston Scientific Co.). The electrical plate (Erbe Nessy® Omega Plate, Erbe Elektromedizin GmbH, Tübingen, Germany) was attached to the sausage for electric current flow. After the insertion of a guidewire and sphincterotome (Tri-Tome PC Protector, Cook Medical Inc., Winston-Salem, NC) into the small tract of the sausage, endoscopic sphincterotomy was successfully performed using an electrosurgical unit (VIO 300D, Erbe Elektromedizin GmbH; Endo cut mode, effect 3, cut duration 2, cut interval 3) (Video 1 shows the implementation of all ERCP procedures using the ERCP phantom).

**Additional file 1: Video 1.** Implementation of ERCP procedures using the ERCP phantom. By changing several modules of the part of ampulla and common bile duct, basic and advanced ERCP procedures are successfully completed.


## Discussion

In contrast to the rapid advances and breakthroughs in endoscopy, ERCP training has not been changed much. Animal models of mainly pigs and dogs were introduced since the beginning [17, 18]. They pose technical difficulties due to the anatomical differences, high cost, and lack of reuse. For overcoming these problems related to animal models, ex-vivo models using some organs from pigs, and hybrid ex-vivo models combining organs from pigs and chickens, have been introduced [19–22]. Although they have been widely used in ERCP training workshops for providing detailed and specific training programs, they have not completely overcome the challenge of anatomical differences, and limitations in their use, especially related to storage and durability. Computer simulators have some advantages as they pose no ethical problems, no storage limitations, and are durable [23–26]. However, they have the disadvantages of not being realistic due to the lack of haptic feedback and high initial-purchase cost. Also, compared with the mechanical method mentioned below, computer simulators do not use real endoscopes or accessories, and have not received much response so far [27]. The mechanical methods involve artificial duodenum, ampulla, and distal bile ducts in a container, so that only some techniques such as cannulation and stent insertion can be practiced [7, 28]. It is simple and portable, and it has been widely used for basic training. However, the training using this model lacks realism, and cannot provide more sophisticated training.

We were trying to find a solution, which would provide a realistic experience during practicing ERCP, before we made our ERCP training silicon model. Although fluoroscopic guidance carries a burden of radiation exposure and space constraints, it is more important to directly feel the ERCP procedure. We also thought that it would be more effective to exercise the procedures while observing the endoscopic and the fluoroscopic images simultaneously. Also, the model would be designed to make repetitive and semi-permanent reuse possible, and not be limited to simple basic ERCP techniques, but to be utilized for all advanced ERCP techniques as much as possible.

In order to make the experience of being placed around the duodenal ampulla and the feeling of using various ERCP accessories as realistic as possible, 3D modeling data were obtained from human CT images, as per previous reported studies [13–15]. One of important steps in this process was to make the stomach and duodenum. We wanted to make a model where the duodenoscope could be inserted in a sequence that followed from esophagus, stomach, and duodenum, but since silicone material was not elastic and stretchable like the human stomach, we decided to cut the stomach for easy insertion of duodenoscope around the duodenal ampulla and omitted the shortening step. Therefore, insertion of the duodenoscope was not difficult, but the direction of the fluoroscopic image had to be inevitably adjusted because of the difference in direction as compared to an actual human (Fig. 5). Another important step in the designing process was to make the ampulla and CBD in various shapes. If this part could be made into various shapes, it could be implemented for various training models and disease models. In order to resolve this issue, we implemented a new 3D printing method to make these parts in various forms, and to connect them to the main body in a module-type assembly format. Another important step was to implement the sphincterotomy model. For the new model to be appealing and used widely in future, it was necessary that it should be more convenient, cost-effective, and reusable than the previous models. As mentioned for other modules, it had to be easily assembled with the main body, electrically operated, easy to store, and relatively inexpensive. Because the present silicone-material phantom utilizes injection molding technique, it can secure many economic benefits in re-production. Since re-production requires only silicone materials and injection molding efforts, we can save the cost of designing for positive phantom model and negative molder model, and the cost of 3D printing of negative molder as well. Therefore, it is possible to reproduce the same phantom at a price of one-third. Because the present phantom has modular configuration, in addition, it can be implemented with minimal efforts of designing and 3D printing for additional variant designs. While looking for suitable material, the curved Vienna sausage met all the conditions for eligibility and could be used in variable sizes and shapes. Also, it is very similar to the practice in the human body, and creating the sphincterotomy model was a very positive and reaffirming experience.

There were some limitations to this study. First, we did not create a model that had all portions of the esophagus, stomach, and duodenum in the same order as humans. We would like to try this procedure again with using silicon with greater flexibility, which is not available currently. Second, transparent silicone is considered to have the advantage of maximizing portability because it does not need fluoroscopic guidance. However, at present, transparent silicone cannot be used for an ERCP training model because the more silicone is transparent, the more it is harder [29]. We hope highly transparent silicon that overcomes these problems would be developed in future. Third, due to the nature of silicone material, the surface tension is higher than that of the bio-tissue. But, if a softener such as glycerin is injected into the tract, this problem can be solved to some extent. Fourth, if part of the intrahepatic bile duct can be made in various shapes, it can be utilized for various disease models. Fifth, if there is a tube-shaped Vienna sausage, we will have more realistic experience with endoscopic sphincterotomy. It can demonstrate procedure-related perforation and allow us to set a safety margin for endoscopic sphincterotomy. Despite these limitations, our silicon model would be a more attractive and professional training model once some 3D technical limitations are resolved. We are certain that ERCP training will be easy and effective if better silicon models are created by overcoming these problems.

## Conclusion

We made a specialized ERCP training silicon model with 3D printing technique. This model is durable, relatively cheap and easy to make, and thus allows the users to perform various specialized ERCP techniques, which increases its chances of being a good ERCP training model.

## Data Availability

The datasets used and/or analyzed during the current study are available from the corresponding author on reasonable request.

## Abbreviations

3D: Three-dimensional
CBD: Common bile duct
CTG: Computed tomography gastrography
ERCP: Endoscopic retrograde cholangiopancreatography
LPO: Left posterior oblique
STL: StereoLithography
